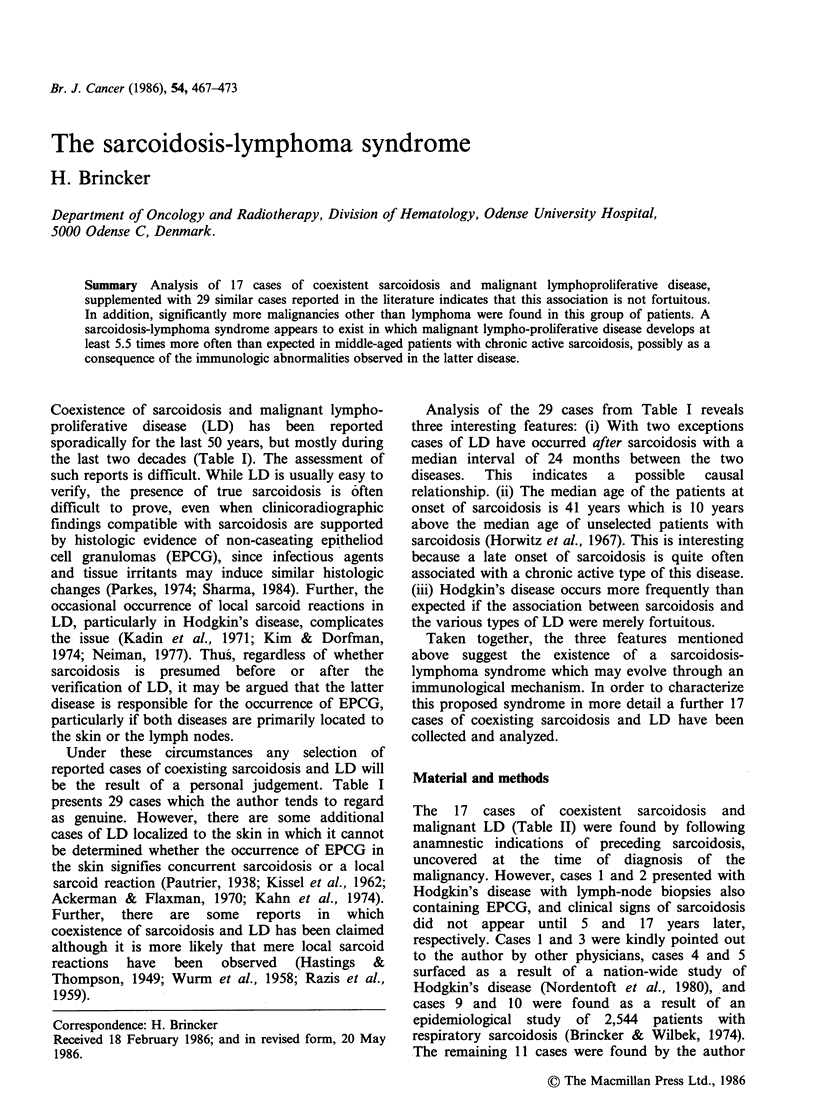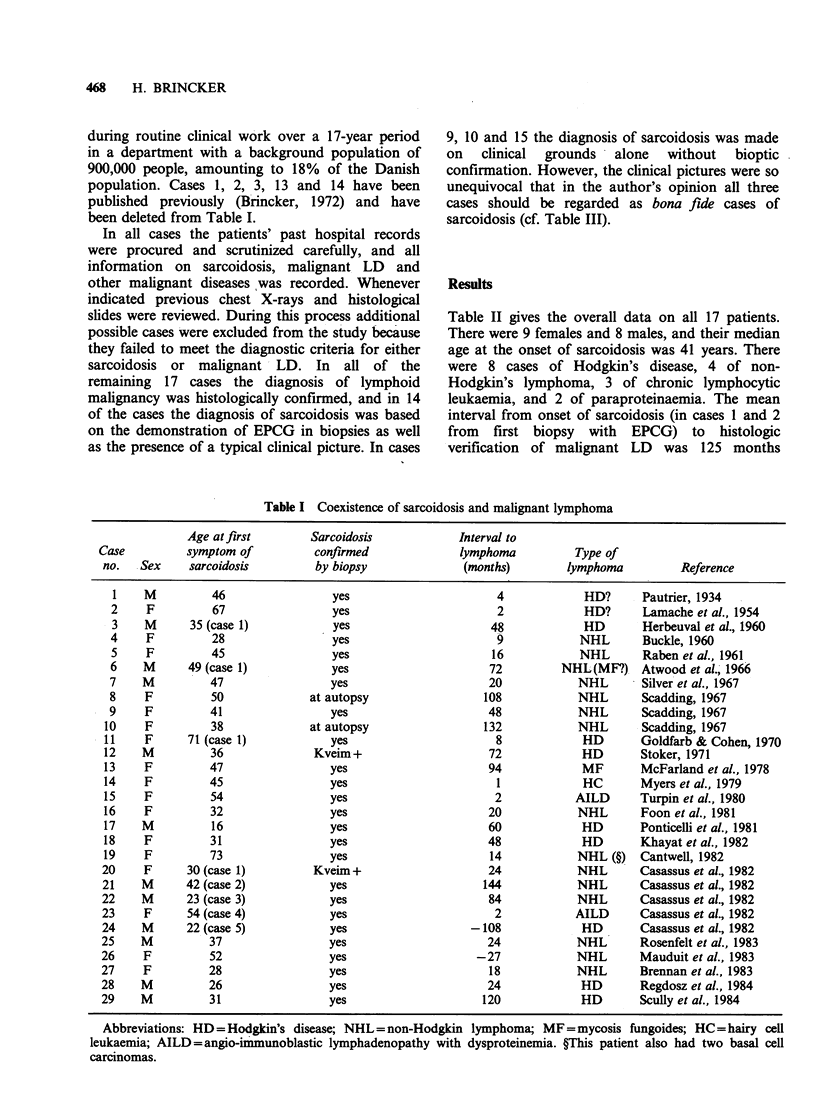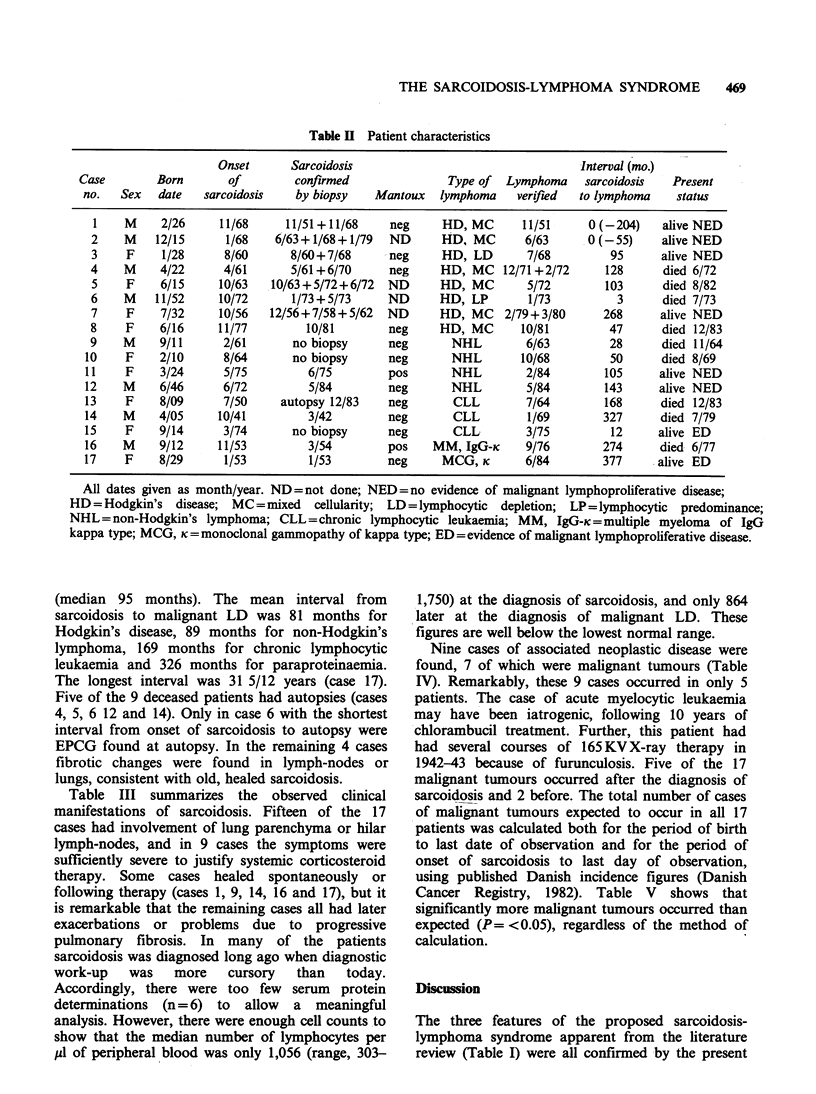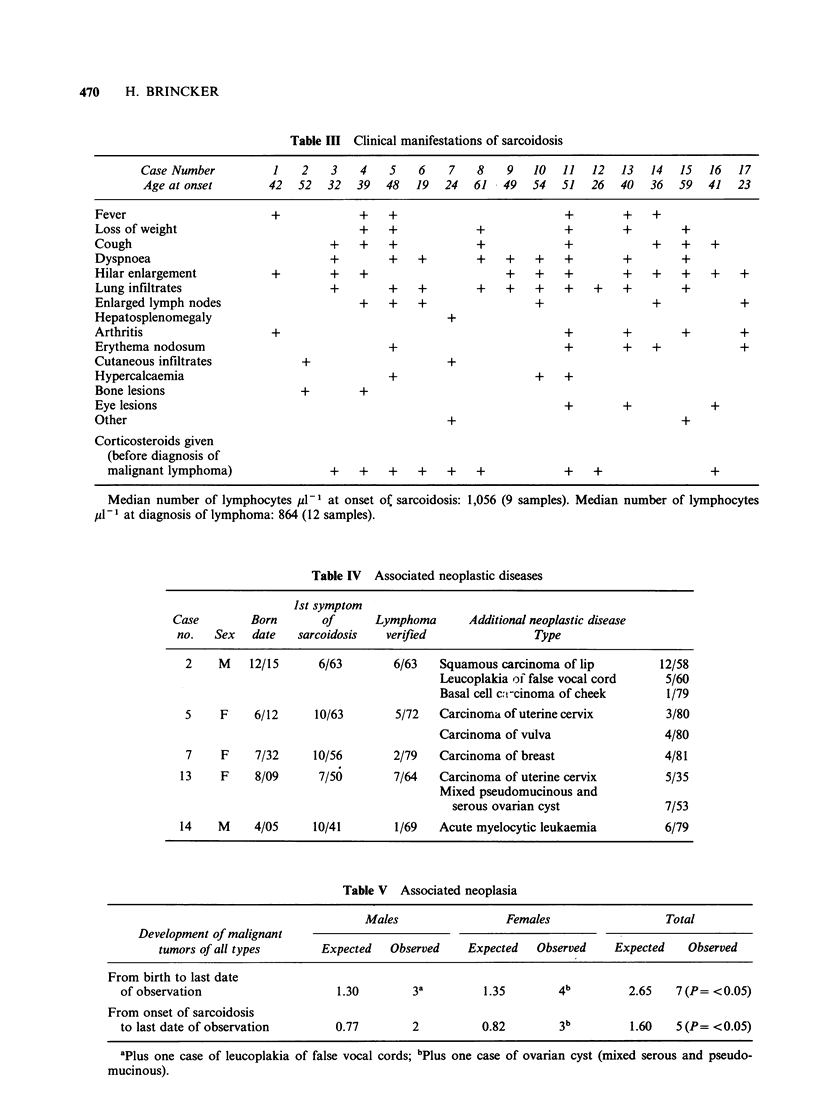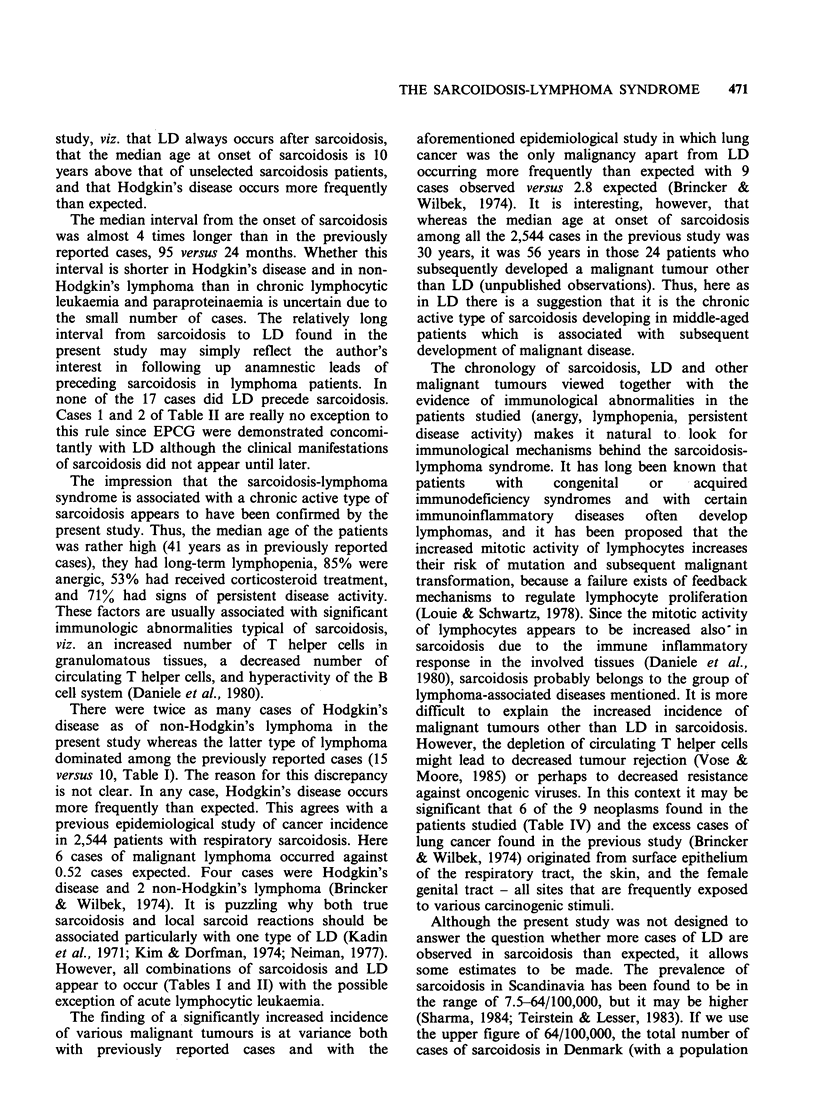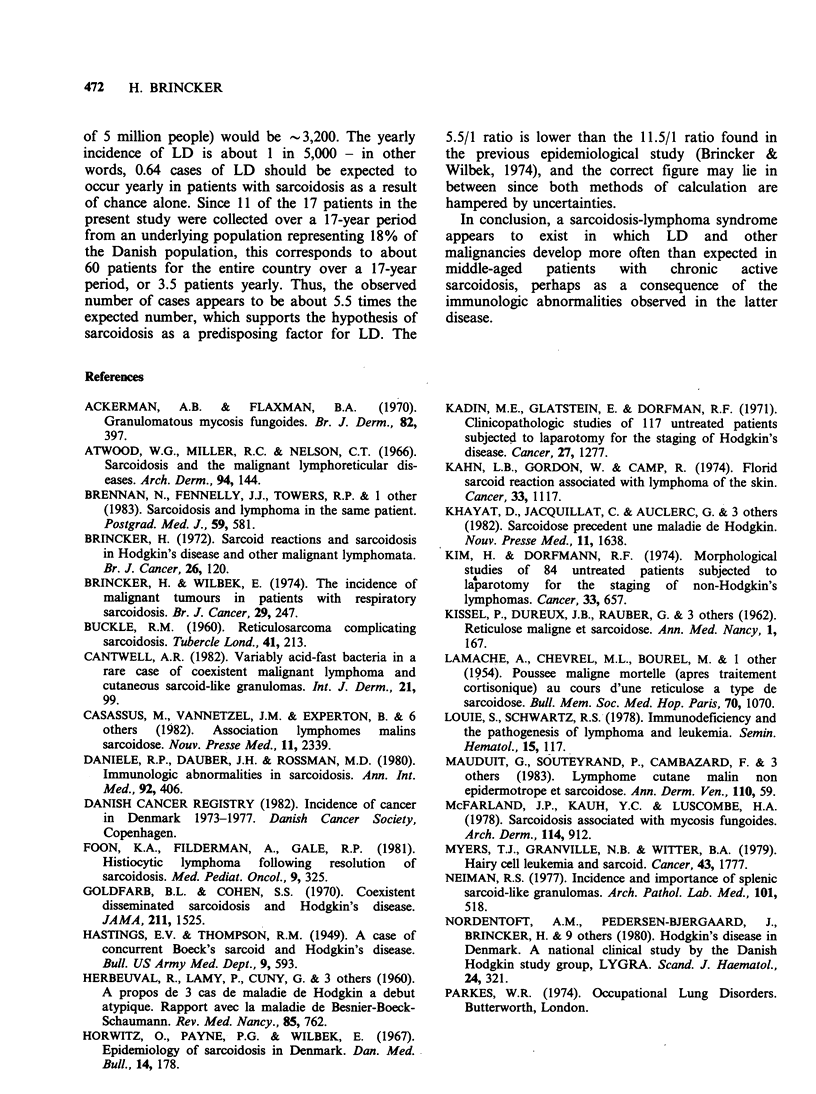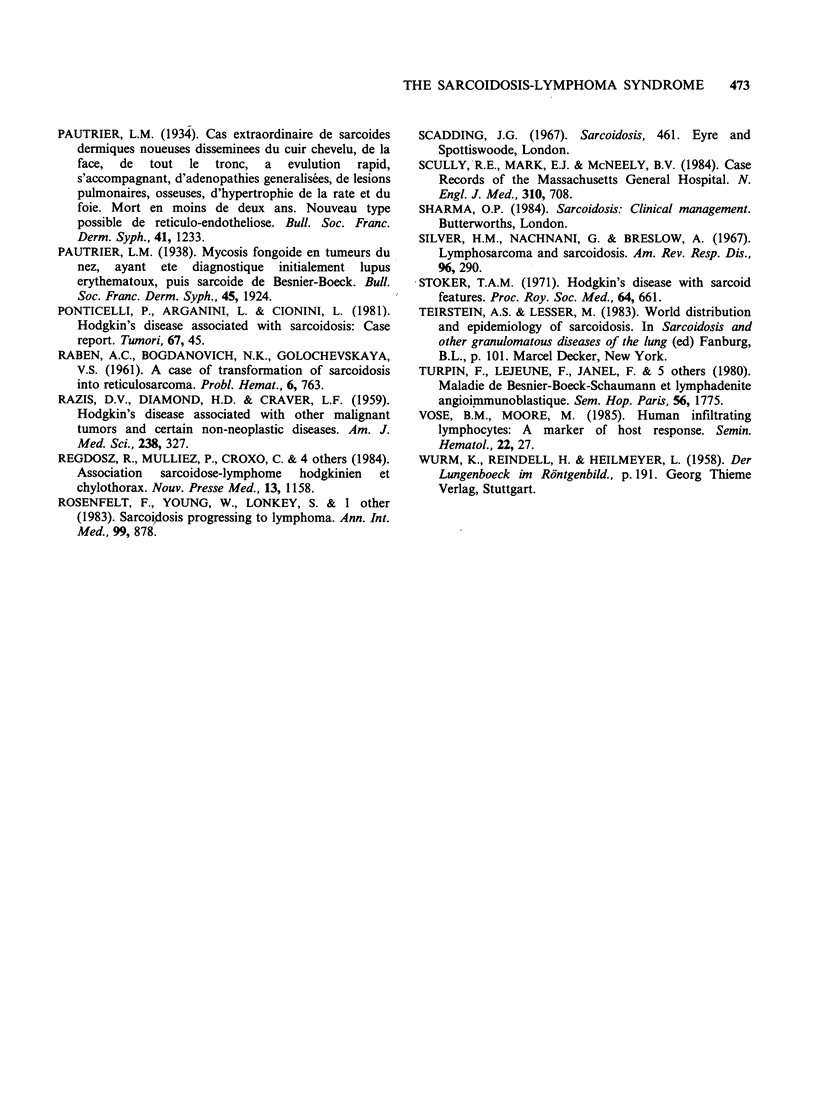# The sarcoidosis-lymphoma syndrome.

**DOI:** 10.1038/bjc.1986.199

**Published:** 1986-09

**Authors:** H. Brincker

## Abstract

Analysis of 17 cases of coexistent sarcoidosis and malignant lymphoproliferative disease, supplemented with 29 similar cases reported in the literature indicates that this association is not fortuitous. In addition, significantly more malignancies other than lymphoma were found in this group of patients. A sarcoidosis-lymphoma syndrome appears to exist in which malignant lympho-proliferative disease develops at least 5.5 times more often than expected in middle-aged patients with chronic active sarcoidosis, possibly as a consequence of the immunologic abnormalities observed in the latter disease.


*Br. J. Cancer* (1986), **54**, 467–473

### The sarcoidosis-lymphoma syndrome

H. Brincker

*Department of Oncology and Radiotherapy, Division of Hematology, Odense University Hospital, 5000 Odense C, Denmark.*

**Summary** Analysis of 17 cases of coexistent sarcoidosis and malignant lymphoproliferative disease, supplemented with 29 similar cases reported in the literature indicates that this association is not fortuitous. In addition, significantly more malignancies other than lymphoma were found in this group of patients. A sarcoidosis-lymphoma syndrome appears to exist in which malignant lympho-proliferative disease develops at least 5.5 times more often than expected in middle-aged patients with chronic active sarcoidosis, possibly as a consequence of the immunologic abnormalities observed in the latter disease.

Coexistence of sarcoidosis and malignant lymphoproliferative disease (LD) has been reported sporadically for the last 50 years, but mostly during the last two decades (Table I). The assessment of such reports is difficult. While LD is usually easy to verify, the presence of true sarcoidosis is often difficult to prove, even when clinicoradiographic findings compatible with sarcoidosis are supported by histologic evidence of non-caseating epitheliod cell granulomas (EPCG), since infectious agents and tissue irritants may induce similar histologic changes (Parkes, 1974; Sharma, 1984). Further, the occasional occurrence of local sarcoid reactions in LD, particularly in Hodgkin's disease, complicates the issue (Kadin *et al.*, 1971; Kim & Dorfman, 1974; Neiman, 1977). Thus, regardless of whether sarcoidosis is presumed before or after the verification of LD, it may be argued that the latter disease is responsible for the occurrence of EPCG, particularly if both diseases are primarily located to the skin or the lymph nodes.

Under these circumstances any selection of reported cases of coexisting sarcoidosis and LD will be the result of a personal judgement. Table I presents 29 cases which the author tends to regard as genuine. However, there are some additional cases of LD localized to the skin in which it cannot be determined whether the occurrence of EPCG in the skin signifies concurrent sarcoidosis or a local sarcoid reaction (Pautrier, 1938; Kissel *et al.*, 1962; Ackerman & Flaxman, 1970; Kahn *et al.*, 1974). Further, there are some reports in which coexistence of sarcoidosis and LD has been claimed although it is more likely that mere local sarcoid reactions have been observed (Hastings & Thompson, 1949; Wurm *et al.*, 1958; Razis *et al.*, 1959).

Correspondence: H. Brincker
Received 18 February 1986; and in revised form, 20 May 1986.

Analysis of the 29 cases from Table I reveals three interesting features: (i) With two exceptions cases of LD have occurred *after* sarcoidosis with a median interval of 24 months between the two diseases. This indicates a possible causal relationship. (ii) The median age of the patients at onset of sarcoidosis is 41 years which is 10 years above the median age of unselected patients with sarcoidosis (Horwitz *et al.*, 1967). This is interesting because a late onset of sarcoidosis is quite often associated with a chronic active type of this disease. (iii) Hodgkin's disease occurs more frequently than expected if the association between sarcoidosis and the various types of LD were merely fortuitous.

Taken together, the three features mentioned above suggest the existence of a sarcoidosis-lymphoma syndrome which may evolve through an immunological mechanism. In order to characterize this proposed syndrome in more detail a further 17 cases of coexisting sarcoidosis and LD have been collected and analyzed.

### Material and methods

The 17 cases of coexistent sarcoidosis and malignant LD (Table II) were found by following anamnestic indications of preceding sarcoidosis, uncovered at the time of diagnosis of the malignancy. However, cases 1 and 2 presented with Hodgkin's disease with lymph-node biopsies also containing EPCG, and clinical signs of sarcoidosis did not appear until 5 and 17 years later, respectively. Cases 1 and 3 were kindly pointed out to the author by other physicians, cases 4 and 5 surfaced as a result of a nation-wide study of Hodgkin's disease (Nordentoft *et al.*, 1980), and cases 9 and 10 were found as a result of an epidemiological study of 2,544 patients with respiratory sarcoidosis (Brincker & Wilbek, 1974). The remaining 11 cases were found by the author

© The Macmillan Press Ltd., 1986

during routine clinical work over a 17-year period in a department with a background population of 900,000 people, amounting to 18% of the Danish population. Cases 1, 2, 3, 13 and 14 have been published previously (Brincker, 1972) and have been deleted from Table I.

In all cases the patients' past hospital records were procured and scrutinized carefully, and all information on sarcoidosis, malignant LD and other malignant diseases was recorded. Whenever indicated previous chest X-rays and histological slides were reviewed. During this process additional possible cases were excluded from the study because they failed to meet the diagnostic criteria for either sarcoidosis or malignant LD. In all of the remaining 17 cases the diagnosis of lymphoid malignancy was histologically confirmed, and in 14 of the cases the diagnosis of sarcoidosis was based on the demonstration of EPCG in biopsies as well as the presence of a typical clinical picture. In cases 9, 10 and 15 the diagnosis of sarcoidosis was made on clinical grounds alone without bioptic confirmation. However, the clinical pictures were so unequivocal that in the author's opinion all three cases should be regarded as *bona fide* cases of sarcoidosis (cf. Table III).

### Results

Table II gives the overall data on all 17 patients. There were 9 females and 8 males, and their median age at the onset of sarcoidosis was 41 years. There were 8 cases of Hodgkin's disease, 4 of non-Hodgkin's lymphoma, 3 of chronic lymphocytic leukaemia, and 2 of paraproteinaemia. The mean interval from onset of sarcoidosis (in cases 1 and 2 from first biopsy with EPCG) to histologic verification of malignant LD was 125 months

**Table I** Coexistence of sarcoidosis and malignant lymphoma

| *Case no.* | *Sex* | *Age at first symptom of sarcoidosis* | *Sarcoidosis confirmed by biopsy* | *Interval to lymphoma (months)* | *Type of lymphoma* | *Reference* |
|---|---|---|---|---|---|---|
| 1 | M | 46 | yes | 4 | HD? | Pautrier, 1934 |
| 2 | F | 67 | yes | 2 | HD? | Lamache *et al.*, 1954 |
| 3 | M | 35 (case 1) | yes | 48 | HD | Herbeuval *et al.*, 1960 |
| 4 | F | 28 | yes | 9 | NHL | Buckle, 1960 |
| 5 | F | 45 | yes | 16 | NHL | Raben *et al.*, 1961 |
| 6 | M | 49 (case 1) | yes | 72 | NHL (MF?) | Atwood *et al.*, 1966 |
| 7 | M | 47 | yes | 20 | NHL | Silver *et al.*, 1967 |
| 8 | F | 50 | at autopsy | 108 | NHL | Scadding, 1967 |
| 9 | F | 41 | yes | 48 | NHL | Scadding, 1967 |
| 10 | F | 38 | at autopsy | 132 | NHL | Scadding, 1967 |
| 11 | F | 71 (case 1) | yes | 8 | HD | Goldfarb & Cohen, 1970 |
| 12 | M | 36 | Kveim + | 72 | HD | Stoker, 1971 |
| 13 | F | 47 | yes | 94 | MF | McFarland *et al.*, 1978 |
| 14 | F | 45 | yes | 1 | HC | Myers *et al.*, 1979 |
| 15 | F | 54 | yes | 2 | AILD | Turpin *et al.*, 1980 |
| 16 | F | 32 | yes | 20 | NHL | Foon *et al.*, 1981 |
| 17 | M | 16 | yes | 60 | HD | Ponticelli *et al.*, 1981 |
| 18 | F | 31 | yes | 48 | HD | Khayat *et al.*, 1982 |
| 19 | F | 73 | yes | 14 | NHL (§) | Cantwell, 1982 |
| 20 | F | 30 (case 1) | Kveim + | 24 | NHL | Casassus *et al.*, 1982 |
| 21 | M | 42 (case 2) | yes | 144 | NHL | Casassus *et al.*, 1982 |
| 22 | M | 23 (case 3) | yes | 84 | NHL | Casassus *et al.*, 1982 |
| 23 | F | 54 (case 4) | yes | 2 | AILD | Casassus *et al.*, 1982 |
| 24 | M | 22 (case 5) | yes | −108 | HD | Casassus *et al.*, 1982 |
| 25 | M | 37 | yes | 24 | NHL | Rosenfelt *et al.*, 1983 |
| 26 | F | 52 | yes | −27 | NHL | Mauduit *et al.*, 1983 |
| 27 | F | 28 | yes | 18 | NHL | Brennan *et al.*, 1983 |
| 28 | M | 26 | yes | 24 | HD | Regdosz *et al.*, 1984 |
| 29 | M | 31 | yes | 120 | HD | Scully *et al.*, 1984 |

Abbreviations: HD = Hodgkin's disease; NHL = non-Hodgkin lymphoma; MF = mycosis fungoides; HC = hairy cell leukaemia; AILD = angio-immunoblastic lymphadenopathy with dysproteinemia. §This patient also had two basal cell carcinomas.

**Table II** Patient characteristics

| *Case no.* | *Sex* | *Born date* | *Onset of sarcoidosis* | *Sarcoidosis confirmed by biopsy* | *Mantoux* | *Type of lymphoma* | *Lymphoma verified* | *Interval (mo.) sarcoidosis to lymphoma* | *Present status* |
|---|---|---|---|---|---|---|---|---|---|
| 1 | M | 2/26 | 11/68 | 11/51 + 11/68 | neg | HD, MC | 11/51 | 0 (−204) | alive NED |
| 2 | M | 12/15 | 1/68 | 6/63 + 1/68 + 1/79 | ND | HD, MC | 6/63 | 0 (−55) | alive NED |
| 3 | F | 1/28 | 8/60 | 8/60 + 7/68 | neg | HD, LD | 7/68 | 95 | alive NED |
| 4 | M | 4/22 | 4/61 | 5/61 + 6/70 | neg | HD, MC | 12/71 + 2/72 | 128 | died 6/72 |
| 5 | F | 6/15 | 10/63 | 10/63 + 5/72 + 6/72 | ND | HD, MC | 5/72 | 103 | died 8/82 |
| 6 | M | 11/52 | 10/72 | 1/73 + 5/73 | ND | HD, LP | 1/73 | 3 | died 7/73 |
| 7 | F | 7/32 | 10/56 | 12/56 + 7/58 + 5/62 | ND | HD, MC | 2/79 + 3/80 | 268 | alive NED |
| 8 | F | 6/16 | 11/77 | 10/81 | neg | HD, MC | 10/81 | 47 | died 12/83 |
| 9 | M | 9/11 | 2/61 | no biopsy | neg | NHL | 6/63 | 28 | died 11/64 |
| 10 | F | 2/10 | 8/64 | no biopsy | neg | NHL | 10/68 | 50 | died 8/69 |
| 11 | F | 3/24 | 5/75 | 6/75 | pos | NHL | 2/84 | 105 | alive NED |
| 12 | M | 6/46 | 6/72 | 5/84 | neg | NHL | 5/84 | 143 | alive NED |
| 13 | F | 8/09 | 7/50 | autopsy 12/83 | neg | CLL | 7/64 | 168 | died 12/83 |
| 14 | M | 4/05 | 10/41 | 3/42 | neg | CLL | 1/69 | 327 | died 7/79 |
| 15 | F | 9/14 | 3/74 | no biopsy | neg | CLL | 3/75 | 12 | alive ED |
| 16 | M | 9/12 | 11/53 | 3/54 | pos | MM, IgG-κ | 9/76 | 274 | died 6/77 |
| 17 | F | 8/29 | 1/53 | 1/53 | neg | MCG, κ | 6/84 | 377 | alive ED |

All dates given as month/year. ND = not done; NED = no evidence of malignant lymphoproliferative disease; HD = Hodgkin's disease; MC = mixed cellularity; LD = lymphocytic depletion; LP = lymphocytic predominance; NHL = non-Hodgkin's lymphoma; CLL = chronic lymphocytic leukaemia; MM, IgG-κ = multiple myeloma of IgG kappa type; MCG, κ = monoclonal gammopathy of kappa type; ED = evidence of malignant lymphoproliferative disease.

(median 95 months). The mean interval from sarcoidosis to malignant LD was 81 months for Hodgkin's disease, 89 months for non-Hodgkin's lymphoma, 169 months for chronic lymphocytic leukaemia and 326 months for paraproteinaemia. The longest interval was 31 5/12 years (case 17). Five of the 9 deceased patients had autopsies (cases 4, 5, 6 12 and 14). Only in case 6 with the shortest interval from onset of sarcoidosis to autopsy were EPCG found at autopsy. In the remaining 4 cases fibrotic changes were found in lymph-nodes or lungs, consistent with old, healed sarcoidosis.

Table III summarizes the observed clinical manifestations of sarcoidosis. Fifteen of the 17 cases had involvement of lung parenchyma or hilar lymph-nodes, and in 9 cases the symptoms were sufficiently severe to justify systemic corticosteroid therapy. Some cases healed spontaneously or following therapy (cases 1, 9, 14, 16 and 17), but it is remarkable that the remaining cases all had later exacerbations or problems due to progressive pulmonary fibrosis. In many of the patients sarcoidosis was diagnosed long ago when diagnostic work-up was more cursory than today. Accordingly, there were too few serum protein determinations (n = 6) to allow a meaningful analysis. However, there were enough cell counts to show that the median number of lymphocytes per μl of peripheral blood was only 1,056 (range, 303–1,750) at the diagnosis of sarcoidosis, and only 864 later at the diagnosis of malignant LD. These figures are well below the lowest normal range.

Nine cases of associated neoplastic disease were found, 7 of which were malignant tumours (Table IV). Remarkably, these 9 cases occurred in only 5 patients. The case of acute myelocytic leukaemia may have been iatrogenic, following 10 years of chlorambucil treatment. Further, this patient had had several courses of 165 KV X-ray therapy in 1942–43 because of furunculosis. Five of the 17 malignant tumours occurred after the diagnosis of sarcoidosis and 2 before. The total number of cases of malignant tumours expected to occur in all 17 patients was calculated both for the period of birth to last date of observation and for the period of onset of sarcoidosis to last day of observation, using published Danish incidence figures (Danish Cancer Registry, 1982). Table V shows that significantly more malignant tumours occurred than expected ($P = < 0.05$), regardless of the method of calculation.

### Discussion

The three features of the proposed sarcoidosis-lymphoma syndrome apparent from the literature review (Table I) were all confirmed by the present

**Table III** Clinical manifestations of sarcoidosis

| *Case Number* | *1* | *2* | *3* | *4* | *5* | *6* | *7* | *8* | *9* | *10* | *11* | *12* | *13* | *14* | *15* | *16* | *17* |
|---|---|---|---|---|---|---|---|---|---|---|---|---|---|---|---|---|---|
| *Age at onset* | *42* | *52* | *32* | *39* | *48* | *19* | *24* | *61* | *49* | *54* | *51* | *26* | *40* | *36* | *59* | *41* | *23* |
| Fever | + | | | + | + | | | | | | + | | + | + | | | |
| Loss of weight | | | | + | + | | | + | | | + | | + | | + | | |
| Cough | | | + | + | + | | | + | | | + | | | + | + | + | |
| Dyspnoea | | | + | | + | + | | + | + | + | + | | + | | + | | |
| Hilar enlargement | + | | + | + | | | | | + | + | + | | + | + | + | + | + |
| Lung infiltrates | | | + | | + | + | | + | + | + | + | + | + | | + | | |
| Enlarged lymph nodes | | | | + | + | + | | | | + | | | | + | | | + |
| Hepatosplenomegaly | | | | | | | + | | | | | | | | | | |
| Arthritis | + | | | | | | | | | | + | | + | | + | | + |
| Erythema nodosum | | | | | + | | | | | | + | | + | + | | | + |
| Cutaneous infiltrates | | + | | | | | + | | | | | | | | | | |
| Hypercalcaemia | | | | | + | | | | | + | + | | | | | | |
| Bone lesions | | + | | + | | | | | | | | | | | | | |
| Eye lesions | | | | | | | | | | | + | | + | | | + | |
| Other | | | | | | | + | | | | | | | | + | | |
| Corticosteroids given (before diagnosis of malignant lymphoma) | | | + | + | + | + | + | + | | | + | + | | | | + | |

Median number of lymphocytes $\mu l^{-1}$ at onset of sarcoidosis: 1,056 (9 samples). Median number of lymphocytes $\mu l^{-1}$ at diagnosis of lymphoma: 864 (12 samples).

**Table IV** Associated neoplastic diseases

| *Case no.* | *Sex* | *Born date* | *1st symptom of sarcoidosis* | *Lymphoma verified* | *Additional neoplastic disease Type* | |
|---|---|---|---|---|---|---|
| 2 | M | 12/15 | 6/63 | 6/63 | Squamous carcinoma of lip | 12/58 |
| | | | | | Leucoplakia of false vocal cord | 5/60 |
| | | | | | Basal cell carcinoma of cheek | 1/79 |
| 5 | F | 6/12 | 10/63 | 5/72 | Carcinoma of uterine cervix | 3/80 |
| | | | | | Carcinoma of vulva | 4/80 |
| 7 | F | 7/32 | 10/56 | 2/79 | Carcinoma of breast | 4/81 |
| 13 | F | 8/09 | 7/50 | 7/64 | Carcinoma of uterine cervix | 5/35 |
| | | | | | Mixed pseudomucinous and serous ovarian cyst | 7/53 |
| 14 | M | 4/05 | 10/41 | 1/69 | Acute myelocytic leukaemia | 6/79 |

**Table V** Associated neoplasia

| *Development of malignant tumors of all types* | *Males* | | *Females* | | *Total* | |
|---|---|---|---|---|---|---|
| | *Expected* | *Observed* | *Expected* | *Observed* | *Expected* | *Observed* |
| From birth to last date of observation | 1.30 | 3[a] | 1.35 | 4[b] | 2.65 | 7 ($P = <0.05$) |
| From onset of sarcoidosis to last date of observation | 0.77 | 2 | 0.82 | 3[b] | 1.60 | 5 ($P = <0.05$) |

[a]Plus one case of leucoplakia of false vocal cords; [b]Plus one case of ovarian cyst (mixed serous and pseudo-mucinous).

study, *viz.* that LD always occurs after sarcoidosis, that the median age at onset of sarcoidosis is 10 years above that of unselected sarcoidosis patients, and that Hodgkin's disease occurs more frequently than expected.

The median interval from the onset of sarcoidosis was almost 4 times longer than in the previously reported cases, 95 *versus* 24 months. Whether this interval is shorter in Hodgkin's disease and in non-Hodgkin's lymphoma than in chronic lymphocytic leukaemia and paraproteinaemia is uncertain due to the small number of cases. The relatively long interval from sarcoidosis to LD found in the present study may simply reflect the author's interest in following up anamnestic leads of preceding sarcoidosis in lymphoma patients. In none of the 17 cases did LD precede sarcoidosis. Cases 1 and 2 of Table II are really no exception to this rule since EPCG were demonstrated concomitantly with LD although the clinical manifestations of sarcoidosis did not appear until later.

The impression that the sarcoidosis-lymphoma syndrome is associated with a chronic active type of sarcoidosis appears to have been confirmed by the present study. Thus, the median age of the patients was rather high (41 years as in previously reported cases), they had long-term lymphopenia, 85% were anergic, 53% had received corticosteroid treatment, and 71% had signs of persistent disease activity. These factors are usually associated with significant immunologic abnormalities typical of sarcoidosis, *viz.* an increased number of T helper cells in granulomatous tissues, a decreased number of circulating T helper cells, and hyperactivity of the B cell system (Daniele *et al.*, 1980).

There were twice as many cases of Hodgkin's disease as of non-Hodgkin's lymphoma in the present study whereas the latter type of lymphoma dominated among the previously reported cases (15 *versus* 10, Table I). The reason for this discrepancy is not clear. In any case, Hodgkin's disease occurs more frequently than expected. This agrees with a previous epidemiological study of cancer incidence in 2,544 patients with respiratory sarcoidosis. Here 6 cases of malignant lymphoma occurred against 0.52 cases expected. Four cases were Hodgkin's disease and 2 non-Hodgkin's lymphoma (Brincker & Wilbek, 1974). It is puzzling why both true sarcoidosis and local sarcoid reactions should be associated particularly with one type of LD (Kadin *et al.*, 1971; Kim & Dorfman, 1974; Neiman, 1977). However, all combinations of sarcoidosis and LD appear to occur (Tables I and II) with the possible exception of acute lymphocytic leukaemia.

The finding of a significantly increased incidence of various malignant tumours is at variance both with previously reported cases and with the aforementioned epidemiological study in which lung cancer was the only malignancy apart from LD occurring more frequently than expected with 9 cases observed *versus* 2.8 expected (Brincker & Wilbek, 1974). It is interesting, however, that whereas the median age at onset of sarcoidosis among all the 2,544 cases in the previous study was 30 years, it was 56 years in those 24 patients who subsequently developed a malignant tumour other than LD (unpublished observations). Thus, here as in LD there is a suggestion that it is the chronic active type of sarcoidosis developing in middle-aged patients which is associated with subsequent development of malignant disease.

The chronology of sarcoidosis, LD and other malignant tumours viewed together with the evidence of immunological abnormalities in the patients studied (anergy, lymphopenia, persistent disease activity) makes it natural to look for immunological mechanisms behind the sarcoidosis-lymphoma syndrome. It has long been known that patients with congenital or acquired immunodeficiency syndromes and with certain immunoinflammatory diseases often develop lymphomas, and it has been proposed that the increased mitotic activity of lymphocytes increases their risk of mutation and subsequent malignant transformation, because a failure exists of feedback mechanisms to regulate lymphocyte proliferation (Louie & Schwartz, 1978). Since the mitotic activity of lymphocytes appears to be increased also in sarcoidosis due to the immune inflammatory response in the involved tissues (Daniele *et al.*, 1980), sarcoidosis probably belongs to the group of lymphoma-associated diseases mentioned. It is more difficult to explain the increased incidence of malignant tumours other than LD in sarcoidosis. However, the depletion of circulating T helper cells might lead to decreased tumour rejection (Vose & Moore, 1985) or perhaps to decreased resistance against oncogenic viruses. In this context it may be significant that 6 of the 9 neoplasms found in the patients studied (Table IV) and the excess cases of lung cancer found in the previous study (Brincker & Wilbek, 1974) originated from surface epithelium of the respiratory tract, the skin, and the female genital tract – all sites that are frequently exposed to various carcinogenic stimuli.

Although the present study was not designed to answer the question whether more cases of LD are observed in sarcoidosis than expected, it allows some estimates to be made. The prevalence of sarcoidosis in Scandinavia has been found to be in the range of 7.5–64/100,000, but it may be higher (Sharma, 1984; Teirstein & Lesser, 1983). If we use the upper figure of 64/100,000, the total number of cases of sarcoidosis in Denmark (with a population of 5 million people) would be ~3,200. The yearly incidence of LD is about 1 in 5,000 – in other words, 0.64 cases of LD should be expected to occur yearly in patients with sarcoidosis as a result of chance alone. Since 11 of the 17 patients in the present study were collected over a 17-year period from an underlying population representing 18% of the Danish population, this corresponds to about 60 patients for the entire country over a 17-year period, or 3.5 patients yearly. Thus, the observed number of cases appears to be about 5.5 times the expected number, which supports the hypothesis of sarcoidosis as a predisposing factor for LD. The 5.5/1 ratio is lower than the 11.5/1 ratio found in the previous epidemiological study (Brincker & Wilbek, 1974), and the correct figure may lie in between since both methods of calculation are hampered by uncertainties.

In conclusion, a sarcoidosis-lymphoma syndrome appears to exist in which LD and other malignancies develop more often than expected in middle-aged patients with chronic active sarcoidosis, perhaps as a consequence of the immunologic abnormalities observed in the latter disease.

**References**

ACKERMAN, A.B. & FLAXMAN, B.A. (1970). Granulomatous mycosis fungoides. *Br. J. Derm.*, **82**, 397.

ATWOOD, W.G., MILLER, R.C. & NELSON, C.T. (1966). Sarcoidosis and the malignant lymphoreticular diseases. *Arch. Derm.*, **94**, 144.

BRENNAN, N., FENNELLY, J.J., TOWERS, R.P. & 1 other (1983). Sarcoidosis and lymphoma in the same patient. *Postgrad. Med. J.*, **59**, 581.

BRINCKER, H. (1972). Sarcoid reactions and sarcoidosis in Hodgkin's disease and other malignant lymphomata. *Br. J. Cancer*, **26**, 120.

BRINCKER, H. & WILBEK, E. (1974). The incidence of malignant tumours in patients with respiratory sarcoidosis. *Br. J. Cancer*, **29**, 247.

BUCKLE, R.M. (1960). Reticulosarcoma complicating sarcoidosis. *Tubercle Lond.*, **41**, 213.

CANTWELL, A.R. (1982). Variably acid-fast bacteria in a rare case of coexistent malignant lymphoma and cutaneous sarcoid-like granulomas. *Int. J. Derm.*, **21**, 99.

CASASSUS, M., VANNETZEL, J.M. & EXPERTON, B. & 6 others (1982). Association lymphomes malins sarcoidose. *Nouv. Presse Med.*, **11**, 2339.

DANIELE, R.P., DAUBER, J.H. & ROSSMAN, M.D. (1980). Immunologic abnormalities in sarcoidosis. *Ann. Int. Med.*, **92**, 406.

DANISH CANCER REGISTRY (1982). Incidence of cancer in Denmark 1973–1977. *Danish Cancer Society*, Copenhagen.

FOON, K.A., FILDERMAN, A., GALE, R.P. (1981). Histiocytic lymphoma following resolution of sarcoidosis. *Med. Pediat. Oncol.*, **9**, 325.

GOLDFARB, B.L. & COHEN, S.S. (1970). Coexistent disseminated sarcoidosis and Hodgkin's disease. *JAMA*, **211**, 1525.

HASTINGS, E.V. & THOMPSON, R.M. (1949). A case of concurrent Boeck's sarcoid and Hodgkin's disease. *Bull. US Army Med. Dept.*, **9**, 593.

HERBEUVAL, R., LAMY, P., CUNY, G. & 3 others (1960). A propos de 3 cas de maladie de Hodgkin a debut atypique. Rapport avec la maladie de Besnier-Boeck-Schaumann. *Rev. Med. Nancy.*, **85**, 762.

HORWITZ, O., PAYNE, P.G. & WILBEK, E. (1967). Epidemiology of sarcoidosis in Denmark. *Dan. Med. Bull.*, **14**, 178.

KADIN, M.E., GLATSTEIN, E. & DORFMAN, R.F. (1971). Clinicopathologic studies of 117 untreated patients subjected to laparotomy for the staging of Hodgkin's disease. *Cancer*, **27**, 1277.

KAHN, L.B., GORDON, W. & CAMP, R. (1974). Florid sarcoid reaction associated with lymphoma of the skin. *Cancer*, **33**, 1117.

KHAYAT, D., JACQUILLAT, C. & AUCLERC, G. & 3 others (1982). Sarcoidose precedent une maladie de Hodgkin. *Nouv. Presse Med.*, **11**, 1638.

KIM, H. & DORFMANN, R.F. (1974). Morphological studies of 84 untreated patients subjected to laparotomy for the staging of non-Hodgkin's lymphomas. *Cancer*, **33**, 657.

KISSEL, P., DUREUX, J.B., RAUBER, G. & 3 others (1962). Reticulose maligne et sarcoidose. *Ann. Med. Nancy*, **1**, 167.

LAMACHE, A., CHEVREL, M.L., BOUREL, M. & 1 other (1954). Poussee maligne mortelle (apres traitement cortisonique) au cours d'une reticulose a type de sarcoidose. *Bull. Mem. Soc. Med. Hop. Paris*, **70**, 1070.

LOUIE, S., SCHWARTZ, R.S. (1978). Immunodeficiency and the pathogenesis of lymphoma and leukemia. *Semin. Hematol.*, **15**, 117.

MAUDUIT, G., SOUTEYRAND, P., CAMBAZARD, F. & 3 others (1983). Lymphome cutane malin non epidermotrope et sarcoidose. *Ann. Derm. Ven.*, **110**, 59.

McFARLAND, J.P., KAUH, Y.C. & LUSCOMBE, H.A. (1978). Sarcoidosis associated with mycosis fungoides. *Arch. Derm.*, **114**, 912.

MYERS, T.J., GRANVILLE, N.B. & WITTER, B.A. (1979). Hairy cell leukemia and sarcoid. *Cancer*, **43**, 1777.

NEIMAN, R.S. (1977). Incidence and importance of splenic sarcoid-like granulomas. *Arch. Pathol. Lab. Med.*, **101**, 518.

NORDENTOFT, A.M., PEDERSEN-BJERGAARD, J., BRINCKER, H. & 9 others (1980). Hodgkin's disease in Denmark. A national clinical study by the Danish Hodgkin study group, LYGRA. *Scand. J. Haematol.*, **24**, 321.

PARKES, W.R. (1974). Occupational Lung Disorders. Butterworth, London.

PAUTRIER, L.M. (1934). Cas extraordinaire de sarcoides dermiques noueuses disseminees du cuir chevelu, de la face, de tout le tronc, a evulution rapid, s'accompagnant, d'adenopathies generalisées, de lesions pulmonaires, osseuses, d'hypertrophie de la rate et du foie. Mort en moins de deux ans. Nouveau type possible de reticulo-endotheliose. *Bull. Soc. Franc. Derm. Syph.*, **41**, 1233.

PAUTRIER, L.M. (1938). Mycosis fongoide en tumeurs du nez, ayant ete diagnostique initialement lupus erythematoux, puis sarcoide de Besnier-Boeck. *Bull. Soc. Franc. Derm. Syph.*, **45**, 1924.

PONTICELLI, P., ARGANINI, L. & CIONINI, L. (1981). Hodgkin's disease associated with sarcoidosis: Case report. *Tumori*, **67**, 45.

RABEN, A.C., BOGDANOVICH, N.K., GOLOCHEVSKAYA, V.S. (1961). A case of transformation of sarcoidosis into reticulosarcoma. *Probl. Hemat.*, **6**, 763.

RAZIS, D.V., DIAMOND, H.D. & CRAVER, L.F. (1959). Hodgkin's disease associated with other malignant tumors and certain non-neoplastic diseases. *Am. J. Med. Sci.*, **238**, 327.

REGDOSZ, R., MULLIEZ, P., CROXO, C. & 4 others (1984). Association sarcoidose-lymphome hodgkinien et chylothorax. *Nouv. Presse Med.*, **13**, 1158.

ROSENFELT, F., YOUNG, W., LONKEY, S. & 1 other (1983). Sarcoidosis progressing to lymphoma. *Ann. Int. Med.*, **99**, 878.

SCADDING, J.G. (1967). *Sarcoidosis*, 461. Eyre and Spottiswoode, London.

SCULLY, R.E., MARK, E.J. & McNEELY, B.V. (1984). Case Records of the Massachusetts General Hospital. *N. Engl. J. Med.*, **310**, 708.

SHARMA, O.P. (1984). *Sarcoidosis: Clinical management.* Butterworths, London.

SILVER, H.M., NACHNANI, G. & BRESLOW, A. (1967). Lymphosarcoma and sarcoidosis. *Am. Rev. Resp. Dis.*, **96**, 290.

STOKER, T.A.M. (1971). Hodgkin's disease with sarcoid features. *Proc. Roy. Soc. Med.*, **64**, 661.

TEIRSTEIN, A.S. & LESSER, M. (1983). World distribution and epidemiology of sarcoidosis. In *Sarcoidosis and other granulomatous diseases of the lung* (ed) Fanburg, B.L., p. 101. Marcel Decker, New York.

TURPIN, F., LEJEUNE, F., JANEL, F. & 5 others (1980). Maladie de Besnier-Boeck-Schaumann et lymphadenite angioimmunoblastique. *Sem. Hop. Paris*, **56**, 1775.

VOSE, B.M., MOORE, M. (1985). Human infiltrating lymphocytes: A marker of host response. *Semin. Hematol.*, **22**, 27.

WURM, K., REINDELL, H. & HEILMEYER, L. (1958). *Der Lungenboeck im Röntgenbild.*, p. 191. Georg Thieme Verlag, Stuttgart.